# Does Combined Reconstruction of the Medial Collateral and Anterior Cruciate Ligaments Provide Better Knee Function? A Systematic Review and Meta-Analysis

**DOI:** 10.3390/jcm13133882

**Published:** 2024-07-01

**Authors:** Károly Csete, Bálint Baráth, Lilla Sándor, Helga Holovic, Péter Mátrai, László Török, Petra Hartmann

**Affiliations:** 1 Department of Traumatology, University of Szeged, 6725 Szeged, Hungary; csete.karoly@med.u-szeged.hu (K.C.); barath.balint@med.u-szeged.hu (B.B.); sandor.lilla.viktoria@med.u-szeged.hu (L.S.); holovichelga98@gmail.com (H.H.); torok.laszlo@med.u-szeged.hu (L.T.); 2 Doctoral School of Multidisciplinary Medical Sciences, University of Szeged, 6720 Szeged, Hungary; 3 Institute for Translational Medicine, University of Pécs, 7624 Pécs, Hungary; matrai.peti@gmail.com; 4 Department of Sports Medicine, University of Szeged, 6725 Szeged, Hungary

**Keywords:** anterior cruciate ligament (ACL) anterior cruciate ligament reconstruction (ACLR), medial collateral ligament (MCL), medial collateral ligament reconstruction (MCLR), International Knee Documentation Committee (IKDC), Lysholm scale, Tegner scale, multiligament knee injury

## Abstract

**Objective:** This study aimed to determine if medial collateral ligament reconstruction (MCLR) alongside anterior cruciate ligament reconstruction (ACLR) preserves knee functionality better than isolated ACLR in combined ACL and MCL tears. **Methods:** MEDLINE, EMBASE, Scopus, CENTRAL, and Web of Science were searched systematically on 31 March 2023. Studies reporting post-operative function after ACLR and ACLR + MCLR in combined injuries were included. Outcomes included International Knee Documentation Committee (IKDC) score, side-to-side difference (SSD), Lysholm, and Tegner scale values. **Results:** Out of 2362 papers, 8 studies met the criteria. The analysis found no significant difference in outcomes (MD = 3.63, 95% CI: [−5.05, 12.3] for IKDC; MD = −0.64, 95% CI: [−3.24, 1.96] for SSD at 0° extension; MD = −1.79, 95% CI: [−4.61, 1.04] for SSD at 30° extension; MD = −1.48, 95% CI: [−16.35, 13.39] for Lysholm scale; MD = −0.21, 95% CI: [−4.29, 3.87] for Tegner scale) between treatments. **Conclusions:** This meta-analysis found no significant difference in outcomes between ACLR and ACLR + MCLR, suggesting that adding MCLR does not provide additional benefits. Due to the heterogeneity and quality of the included studies, further high-quality randomized controlled trials are needed to determine the optimal treatment for combined severe MCL–ACL injuries.

## 1. Introduction

The treatment of combined anterior cruciate ligament (ACL) and medial collateral ligament (MCL) injuries has evolved over the past 30 years, and early ACL reconstruction and acute MCL repair are recommended when there is increased medial joint space opening with valgus stress in extension, a significant meniscotibial deep MCL injury, or a high-riding medial meniscus [1]. However, some authors have observed better outcomes with late ACL reconstruction compared to early reconstruction [2]. In addition, a study on 19,457 patients from the Swedish National Knee Ligament Registry found that non-surgical treatment of a concomitant MCL injury increased the risk of ACL revision [3]. Therefore, the optimal treatment for combined ACL and MCL injuries is still controversial, and there is no up-to-date consensus regarding the superiority of nonoperative versus operative management in severe (Grade III) MCL tears of combined ACL-MCL injuries [4]. However, some studies have suggested that early medial reconstruction combined with severely injured MCLs can decrease residual medial laxity in ACL reconstruction [5]. 

The biomechanical synergy between the ACL and MCL plays a crucial role in knee stability, and damage to both ligaments can result in significant functional impairment and instability. This intricate relationship highlights the importance of a comprehensive approach to treatment that addresses both ligaments to restore knee function effectively [6]. 

The treatment strategy for combined ACL/MCL injuries largely depends on the severity of the MCL tear, which significantly influences management decisions [7,8]. For partial MCL injuries (Grade I or II), non-operative treatment is usually recommended for the MCL. During this period, the knee is allowed to heal, and normal range of motion is restored. Once the MCL has healed, ACL reconstruction is performed [8]. Studies have consistently shown that this approach is effective, as it minimizes the risk of complications and promotes the optimal healing of both ligaments. 

However, the treatment of combined ACL and Grade III MCL injuries is more controversial. Some studies advocate for conservative management, suggesting that with aggressive physical therapy following a brief immobilization period, patients can achieve favorable outcomes. In an early study, 68% of patients treated non-operatively for severe ACL/Grade III MCL injuries were able to return to their original activity levels after long-term follow-up [9]. In contrast, other studies indicate that the non-operative management of Grade III MCL injuries is less successful and can contribute to chronic knee instability and increased stress on a reconstructed ACL [3,10]. This has led to a preference for surgical intervention with the aim of restoring stability and function effectively. Surgical options range from primary MCL repair to anatomic reconstruction using various grafts, including tibialis anterior allograft, Achilles tendon allograft, iliotibial band autograft, or hamstring tendon autograft [11,12,13,14]. Despite these advancements, the clinical decision-making process remains complex, often influenced by factors such as the severity of ligament damage, patient-specific considerations, and surgeon expertise. It should be noted that five previous systematic reviews have tried to explore this topic, each with limitations [15,16,17,18]. In 2010, Papas et al. first attempted to provide evidence on the treatment of combined ACL + MCL injuries, but the included studies had too much heterogeneity in terms of patient characteristics and management methods to endorse any of treatments [15]. In the comprehensive study by Grant et al., there was inconsistency in the way that standard outcomes were reported and there was variability in the definition/diagnosis of a Grade III MCL tear [16]. Since then, new clinical studies have also been published [5,6,19,20], including a registry data analysis with a large but non-selective study population [19]. Two new reviews have been published since then: Rao et al. documented heterogeneous outcome measures and varying follow-up times [17], and in a recent analysis there was a high variability of surgical techniques and outcome reporting [18].

In conclusion, the optimal treatment for these injuries is still debated, with some studies suggesting that repair of the MCL can achieve satisfactory outcomes, while others advocate for the reconstruction of both ligaments using autografts or allografts. Therefore, the aim of this systematic review was to evaluate, compare, and update the evidence as to whether MCL reconstruction (MCLR) and ACL reconstruction (ACLR) will result in better maintained knee functionality than isolated ACLR following combined ACL and severe MCL tearing.

## 2. Materials and Methods

### 2.1. Protocol and Registration

This meta-analysis was conducted in accordance with the PRISMA (Preferred Reporting Items for Systematic Reviews and Meta-Analyses) guidelines [21]. The study protocol was registered in the international prospective register of systematic reviews, PROSPERO (CRD42022380084), prior to data extraction.

### 2.2. Eligibility Criteria

In this study, randomized controlled trials (RCTs) and observational studies were included. Articles reporting post-operative function following ACLR and ACLR + MCLR (diagnosed by physical examination, arthroscopy, or MRI) in combined injury were deemed eligible. Studies that were written in a language other than English, or investigated patients with bilateral ACL insufficiency, partial ACL tear, mild MCL tear (Grade I or II), a history of previous ligament surgery or posterior cruciate ligament (PCL) injury, open injuries, diagnosis of concomitant fracture or avulsion injury, and patients under 18 years of age were excluded. 

### 2.3. Information Sources and Literature Search

MEDLINE, EMBASE, Scopus, the CENTRAL, and Web of Science databases were searched without restrictions, using the following phrases with MeSH keywords: (“Medial Collateral Ligament, Knee” [Mesh]) AND (“Anterior Cruciate Ligament” [Mesh] OR “Hamstring Tendons” [Mesh] OR “Anterior Cruciate Ligament Reconstruction” [Mesh] OR “Bone-Patellar Tendon-Bone Grafts” [Mesh] OR “Bone-Patellar Tendon-Bone Grafting” [Mesh]). For CENTRAL, EMBASE, Scopus, and Web of Science, the search terms were adapted correspondingly.

### 2.4. Study Selection

**Figure 1:**
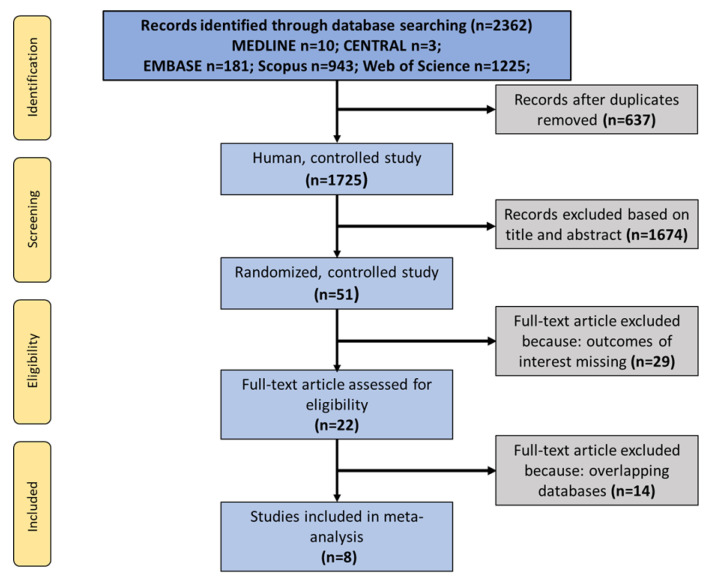
PRISMA flowchart of a search strategy with inclusions and exclusions.

All articles detected by our systematic search were imported into an EndNote file. As a first step, duplicates were sorted out. The remaining articles were screened independently by two review authors (L.S. and B.B.) based on the inclusion criteria outlined above (Figure 1). Disagreements between them over the eligibility of particular studies were resolved through discussion with a third reviewer (K.C.).

### 2.5. Data Extraction

Extracted information includes the study setting, study population, participant demographics and baseline characteristics, details of the intervention and control conditions, study methodology, recruitment and study completion rates, outcomes, and times of measurement. Objective parameters were selected as primary outcome measures to assess knee function, such as the International Knee Documentation Committee (IKDC) score, which is based on physical examination and evaluates symptoms, range of motion and knee laxity [22], and side-to-side difference (SSD) in antero-posterior displacement of the tibia when comparing the two knees. Secondary outcomes included subjective functional scores, such as the patient-reported Lysholm and Tegner scale [23,24].

### 2.6. Assessment of Study Quality and Risk of Bias

The two review authors (L.S. and B.B.) independently assessed the risk of bias in the included studies. Disagreements over the risk of bias in particular studies were resolved by discussion, involving a third review author. The risk of bias assessment of non-randomized controlled trials was performed based on the Cochrane risk of bias tool, checking missing data, internal data consistency, and randomization integrity.

### 2.7. Data Synthesis and Statistical Analysis

The statistical analyses were performed with R (R Core Team 2022, v4.2.0). For calculations and plots, we used the meta package (Schwarzer 2022, v6.0.0). For the continuous outcomes, the mean difference with 95% CI was calculated as the effect size. The values extracted to estimate the mean difference and its variance were the sample size, the mean, the standard deviation, and the minimum and maximum values in the two groups, if available. The random effect model was used to summarize the mean differences. Statistical heterogeneity across trials was assessed by means of the Cochrane Q test and the I-squared values [25]. Forest plots were used to graphically summarize the results. Where applicable, we reported the prediction intervals (i.e., the expected range of effects of future studies), following the recommendations of IntHout et al. [26,27]. The results of meta-analysis may be limited by the selection of an incomplete set of studies, the presence of studies with small sample sizes, and the heterogeneity of methods used in the studies. The statistical methods of the articles used in the meta-analysis were also different; thus, we had less data for each parameter [28].

## 3. Results

### 3.1. Study Selection and Characteristics

A total of 2362 articles were obtained through our search strategy on 31 March 2023. After excluding duplicates, 1725 articles were screened for eligibility based on the title and abstract. A total of 51 studies were considered for inclusion; however, upon further screening, an additional 29 articles were excluded that did not contain outcomes of interest, and 14 more to avoid data overlapping. Ultimately a total of 8 studies were included in the final analyses [5,6,12,19,20,29,30,31] (Figure 1). A total of 25,577 patients were included; their demographics are shown in Table 1. 

**Table 1:** Demographics and characteristics of included studies.

Author, Year	Design	Country	Recruitment Period	Groups	Patients’ Characteristics					
					Patient	Age (y)		Gender		BMI
						Mean	SD	Male (%)	Female (%)	Mean
Nakamura, 2003 [12]	Prospective	Japan	1995–1997	ACLR	17	22.8	ND	85.9	14.1	ND
				ACLR + MCL		22.8	ND	81.4	18.6	ND
Lutz, 2021 [6]	Retrospective	Germany	2014–2019	ACLR	40	33	8	60	20	27.4
				ACLR + MCL		40	12	40	80	25
Halinen, 2006 [29]	Prospective	Finland	1996–2001	ACLR	47	38.3	ND	50	50	ND
				ACLR + MCL		40.3	ND	34.8	65.2	ND
Zaffagnini, 2011 [30]	Retrospective	Italy	2005	ACLR	51	34	8.4	96.9	3.1	ND
				ACLR + MCL		38	14.7	94.7	44	ND
Westermann, 2019 [31]	Retrospective	USA	2002–2008	ACLR	27	27.9	ND	45	55	27.9
				ACLR + MCL		27.9	ND	56	44	27.9
Sim, 2021 [5]	Retrospective	Korea	2008–2017	ACLR	105	33.1	12.4	86.8	13.2	24.8
				ACLR + MCL		33.1	12.4	86.8	13.2	24.8
Lind, 2020 [19]	Retrospective	Denmark	2005–2016	ACLR	25,282	28.3	ND	61	39	ND
				ACLR + MCL		33.2	ND	70	30	ND
Funchal, 2018 [20]	Prospective	Brazil	2004–2016	ACLR	112	32.5	ND	77	23	ND
				ACLR + MCL		29.7	ND	77	23	ND

ND: No data about this parameter.

### 3.2. Risk of Bias Assessment

**Figure 2:**
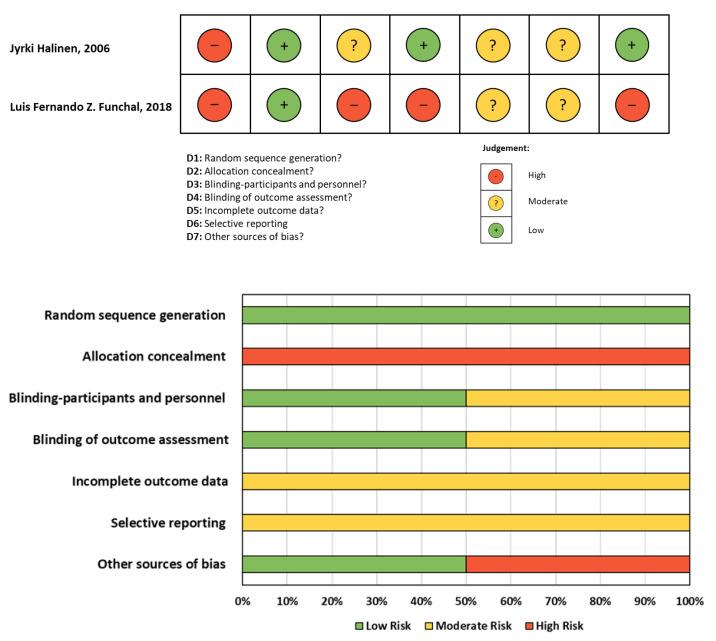
Risk Of Bias (ROB2) assessment of included RCT studies [20,29].

**Figure 3:**
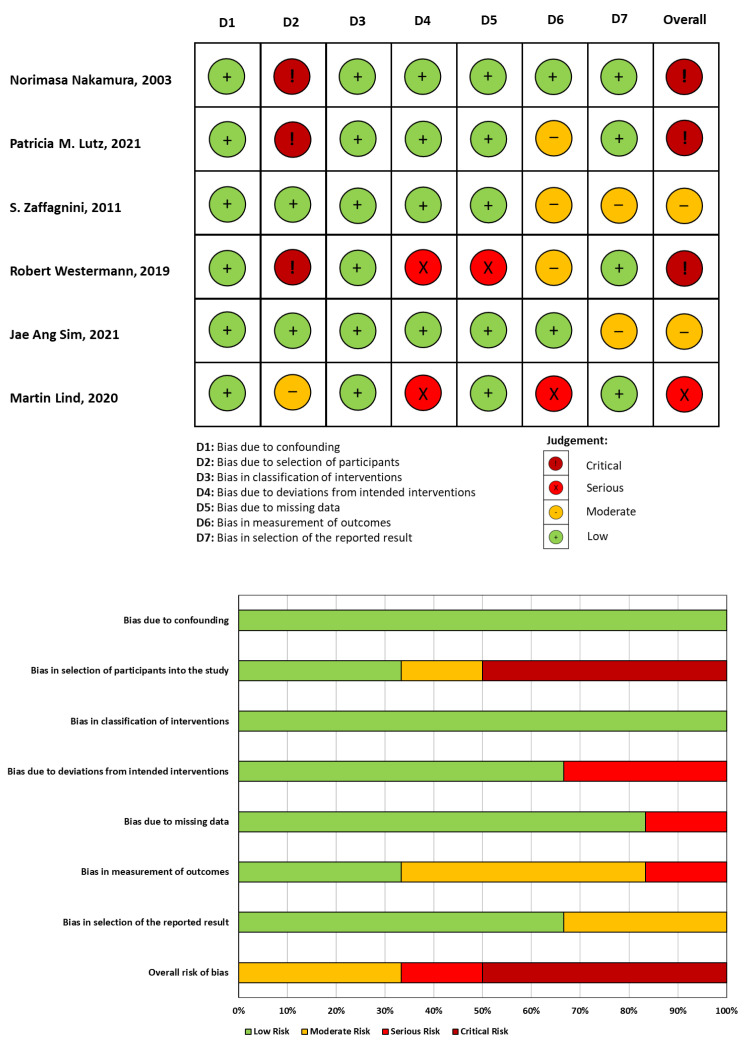
Risk of Bias in Non-Randomised Studies of Interventions (ROBINS-I) assessment of included non-RCT studies [5,6,12,19,30,31].

The Cochrane Risk of Bias tool was used to assess the risk of bias for each study. The Cochrane Risk of Bias tool for randomized trials (ROB2) was applied to RCT studies (Figure 2), while the Cochrane Risk Of Bias in Non-Randomised Studies of Interventions (ROBINS-I) was used for non-RCT studies (Figure 3). The studies included in the analysis were judged to have a moderate to high risk, primarily attributed to selection criteria and detection bias.

### 3.3. Primary Outcomes: IKDC and SSD

**Figure 4:**
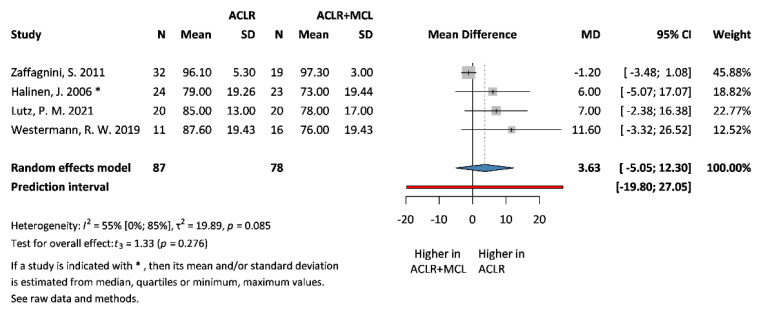
**Forest plot comparing IKDC scores following ACLR + MCL vs. ACLR.** Squares represent individual study effects, with the size of the square indicating the weight of the study in the meta-analysis. The diamond represents the summary effect from meta-analysis. Horizontal bars denote the 95% CIs. There is no evidence of small study effects in the test or the formal plot. MD: mean difference; SD: standard deviation; CI: confidence interval [6,29,30,31].

**Figure 5:**
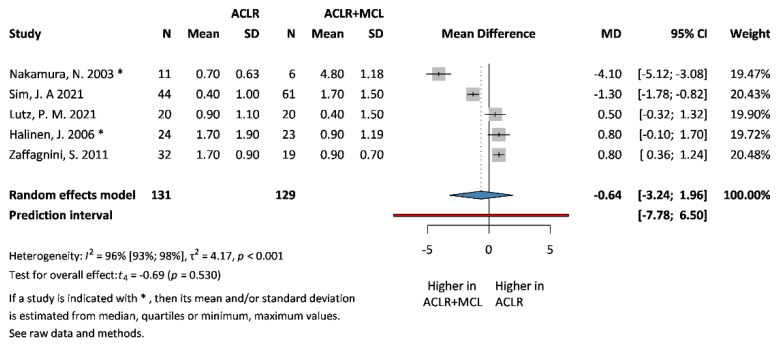
**Forest plot comparing SSD scores at 0° extension following ACLR + MCL vs. ACLR.** Squares represent individual study effects, with the size of the square indicating the weight of the study in the meta-analysis. The diamond represents the summary effect from the meta-analysis. Horizontal bars denote the 95% CIs. There is no evidence of small study effects in the test or the formal plot. MD: mean difference; SD: standard deviation; CI: confidence interval [5,6,12,29,30].

**Figure 6:**
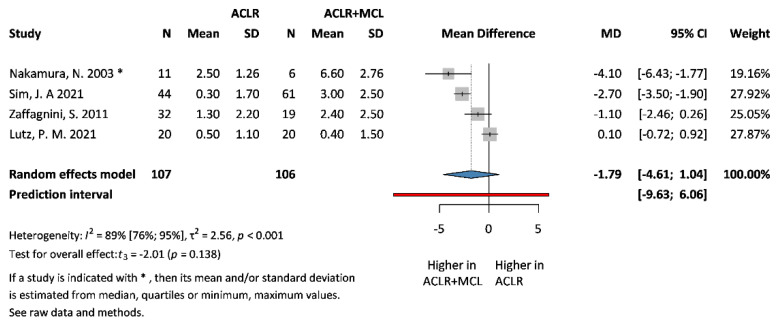
**Forest plot comparing SSD scores at 30° extension following ACLR + MCL vs. ACLR.** Squares represent individual study effects, with the size of the square indicating the weight of the study in the meta-analysis. The diamond represents the summary effect from the meta-analysis. Horizontal bars denote the 95% CIs. There is no evidence of small study effects in the test or the formal plot. MD: mean difference; SD: standard deviation; CI: confidence interval [5,6,12,30].

We investigated the difference in outcomes in IKDC and SSD scores following ACLR or ACLR + MCLR. Four studies with available IKDC scores were selected for analysis, covering a total of 165 patients. The mean difference (MD, the pooled effect size) was 3.63 between ACLR and ACLR + MCLR groups. The 95% confidence interval (CI) of MD was −5.05 to 12.3, indicating that the effect size in the comparable studies could fall within this range (Figure 4). For SSD at 0°, five studies were selected for analysis, covering a total of 260 patients. The MD (the pooled effect size) was −0.64 between ACLR and ACLR + MCLR groups. The 95% CI of MD was −3.24 to 1.96 (Figure 5). For SSD at 30°, four studies were selected for analysis, covering a total of 213 patients. The MD was −1.79 between ACLR and ACLR + MCLR groups. The 95% CI of MD was −4.61 to 1.04 (Figure 6). In all three cases, the CI of pooled effect size includes the value 0, suggesting that there is no statistically significant difference in the effect between the two groups.

### 3.4. Secondary Outcomes: Lysholm and Tegner Scale

**Figure 7:**
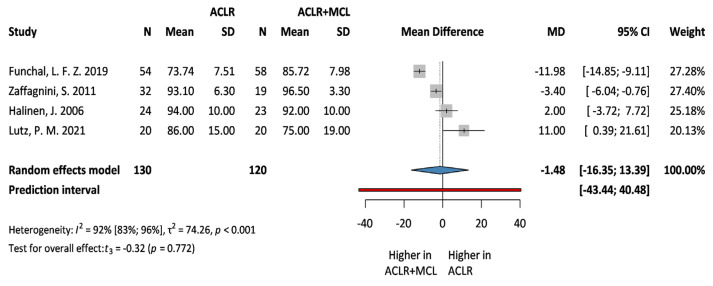
**Forest plot comparing Lysholm scale following ACLR + MCL vs. ACLR.** Squares represent individual study effects, with the size of the square indicating the weight of the study in the meta-analysis. The diamond represents the summary effect from the meta-analysis. Horizontal bars denote the 95% CIs. There is no evidence of small study effects in the test or the formal plot. MD: mean difference; SD: standard deviation; CI: confidence interval [6,20,29,30].

**Figure 8:**
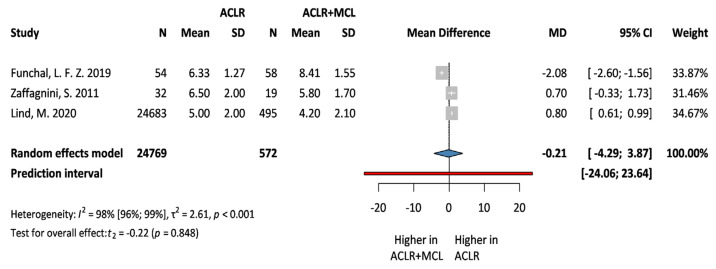
**Forest plot comparing Tegner scale following ACLR + MCL vs. ACLR.** Squares represent individual study effects, with the size of the square indicating the weight of the study in the meta-analysis. The diamond represents the summary effect from the meta-analysis. Horizontal bars denote the 95% CIs. There is no evidence of small study effects in the test or the formal plot. MD: mean difference; SD: standard deviation; CI: confidence interval [19,20,30].

Four studies were included in the analysis of Lysholm scales, covering a total of 250 patients. The MD was −1.48 between ACLR and ACLR + MCL groups. The 95% CI of MD was −16.35 to 13.39 (Figure 7). For Tegner scales, three studies were included in the analysis, covering a total of 25,341 patients. The MD (the pooled effect size) was −0.21 between ACLR and ACLR + MCL groups. The 95% CI of MD was −4.29 to 3.87 (Figure 8). Similarly to the primary outcomes, the CI of the pooled effect size includes the value 0, indicating that there is no statistically significant difference in the effect between the two groups.

## 4. Discussion

Combined ACL and MCL injury is the most common type of multiligament knee injury. The optimal treatment for these injuries is still debated. Some studies suggest that MCL repair can achieve satisfactory outcomes, while others advocate for the reconstruction of both ligaments using autografts or allografts.

The results of this meta-analysis indicated no significant difference between ACLR and the combined reconstruction of the ACL and severe MCL tears regarding knee stability, function, or quality of life. This conclusion is drawn from the analysis of objective primary and subjective secondary outcomes of knee function.

The IKDC scores in this analysis, encompassing 165 patients, showed no significant difference between isolated ACLR and combined ACLR + MCLR. This finding aligns with previous studies, such as those by Tischer et al. [32] and Yang et al. [33], who also found no significant improvement in IKDC scores with combined reconstruction. These studies suggest that the addition of MCLR does not provide substantial benefits in terms of knee function, as measured by the IKDC scale. Similarly, the SSD values from five studies indicated no significant difference between the two groups. This finding is consistent with previous research [18,19], which reported similar outcomes in anterior–posterior knee stability as to whether MCLR was added or not.

The results for the Lysholm scale in our analysis showed no significant difference in subjective knee function and symptoms between the two treatment approaches. Previous studies also concluded that patient-reported outcomes and subjective knee functions do not improve significantly with the addition of MCLR to ACLR [34,35]. The Tegner scale results also indicated no significant difference, consistent with clinical studies that found no significant improvement in returns to sports and activity levels with combined reconstruction [18,36].

However, the quality of evidence was moderate, and there was relatively high heterogeneity among the studies. Therefore, these findings should be interpreted with caution, and further randomized controlled trials are needed to confirm the optimal treatment for combined MCL–ACL injuries. 

The choice of treatment for combined MCL–ACL injuries should be individualized based on several factors, including the severity and chronicity of the MCL injury, the patient’s activity level and expectations, and the surgeon’s preference and experience [37,38,39,40,41]. Repair of the MCL may offer advantages such as preserving native tissue and avoiding graft-related complications, while reconstructing the MCL may provide benefits such as restoring normal knee joint anatomy and biomechanics and improving rotational stability [17,42]. Both treatment options can be performed concurrently with ACL reconstruction using autografts or allografts. 

This study has several limitations that should be acknowledged. First, the inclusion criteria were broad and excluded studies with mild (Grade I-II) MCL lesions, but did not further distinguish between different types of MCL lesions. Second, the outcome measures were heterogeneous and not standardized across the studies. Third, the follow-up periods varied widely and were insufficiently long to adequately assess long-term outcomes or complications. Fourth, there was a risk of publication bias and selection bias due to the absence of randomized controlled trials and prospective comparative studies. Fifth, data on patient satisfaction, return to sports, or cost-effectiveness were lacking.

## 5. Conclusions

The results of this meta-analysis support the current consensus in the medical literature: isolated ACLR effectively treats combined ACL and severe (Grade III) MCL injuries. The findings suggest that conservative treatment can lead to the satisfactory healing of MCL injuries, and additional MCLR does not provide significant functional benefits. This aligns with findings from multiple studies indicating that combined reconstruction may not always be necessary, and isolated ACLR can be a viable treatment option for these injuries.

However, due to the heterogeneity and low quality of the included studies, these results should be interpreted cautiously. Further high-quality randomized controlled trials are necessary to establish the optimal treatment approach for combined severe MCL–ACL injuries.

## Data Availability

The data that support the findings of this study are available from the corresponding author upon reasonable request.

## References

[1] Tandogan N.R., Kayaalp A. (2016). Surgical treatment of medial knee ligament injuries: Current indications and techniques. EFORT Open Rev..

[2] Millett P.J., Pennock A.T., Sterett W.I., Steadman J.R. (2004). Early ACL Reconstruction in Combined ACL—MCL Injuries. J. Knee Surg..

[3] Svantesson E., Senorski E.H., Alentorn-Geli E., Westin O., Sundemo D., Grassi A., Čustović S., Samuelsson K. (2019). Increased risk of ACL revision with non-surgical treatment of a concomitant medial collateral ligament injury: A study on 19,457 patients from the Swedish National Knee Ligament Registry. Knee Surg. Sports Traumatol. Arthrosc..

[4] Shultz C.L., Poehlein E., Morriss N.J., Green C.L., Hu J., Lander S., Amoo-Achampong K., Lau B.C. (2024). Nonoperative Management, Repair, or Reconstruction of the Medial Collateral Ligament in Combined Anterior Cruciate and Medial Collateral Ligament Injuries—Which Is Best? A Systematic Review and Meta-analysis. Am. J. Sports Med..

[5] Sim J.A., Na Y.G., Choi J.W., Lee B.H. (2021). Early medial reconstruction combined with severely injured medial collateral ligaments can decrease residual medial laxity in anterior cruciate ligament reconstruction. Arch. Orthop. Trauma. Surg..

[6] Lutz P.M., Höher L.S., Feucht M.J., Neumann J., Junker D., Wörtler K., Imhoff A.B., Achtnich A. (2021). Ultrasound-based evaluation revealed reliable postoperative knee stability after combined acute ACL and MCL injuries. J. Exp. Orthop..

[7] Mangine R.E., Minning S.J., Eifert-Mangine M., Colosimo A.J., Donlin M. (2008). Management of the Patient with an ACL/MCL Injured Knee. N. Am. J. Sports Phys. Ther..

[8] Smyth M.P., Koh J.L. (2015). A Review of Surgical and Nonsurgical Outcomes of Medial Knee Injuries. Sports Med. Arthrosc. Rev..

[9] Jokl P., Kaplan N., Stovell P., Keggi K. (1984). Non-operative treatment of severe injuries to the medial and anterior cruciate ligaments of the knee. J. Bone Joint Surg..

[10] Marx R.G., Hetsroni I. (2012). Surgical Technique: Medial Collateral Ligament Reconstruction Using Achilles Allograft for Combined Knee Ligament Injury. Clin. Orthop. Relat. Res..

[11] Dong J., Wang X.F., Men X., Zhu J., Walker G.N., Zheng X.Z., Gao J.B., Chen B., Wang F., Zhang Y. (2015). Surgical Treatment of Acute Grade III Medial Collateral Ligament Injury Combined with Anterior Cruciate Ligament Injury: Anatomic Ligament Repair Versus Triangular Ligament Reconstruction. Arthrosc. J. Arthrosc. Relat. Surg..

[12] Nakamura N., Horibe S., Toritsuka Y., Mitsuoka T., Yoshikawa H., Shino K. (2003). Acute Grade III Medial Collateral Ligament Injury of the Knee Associated with Anterior Cruciate Ligament Tear: The Usefulness of Magnetic Resonance Imaging in Determining a Treatment Regimen. Am. J. Sports Med..

[13] Yoshiya S., Kuroda R., Mizuno K., Yamamoto T., Kurosaka M. (2005). Medial Collateral Ligament Reconstruction Using Autogenous Hamstring Tendons. Am. J. Sports Med..

[14] Zhang H., Sun Y., Han X., Wang Y., Wang L., Alquhali A., Bai X. (2014). Simultaneous Reconstruction of the Anterior Cruciate Ligament and Medial Collateral Ligament in Patients with Chronic ACL-MCL Lesions. Am. J. Sports Med..

[15] Papalia R., Osti L., Del Buono A., Denaro V., Maffulli N. (2010). Management of combined ACL-MCL tears: A systematic review. Br. Med. Bull..

[16] Grant J.A., Tannenbaum E., Miller B.S., Bedi A. (2012). Treatment of Combined Complete Tears of the Anterior Cruciate and Medial Collateral Ligaments. Arthrosc. J. Arthrosc. Relat. Surg..

[17] Rao R., Bhattacharyya R., Andrews B., Varma R., Chen A. (2022). The management of combined ACL and MCL injuries: A systematic review. J. Orthop..

[18] Wright M.L., Coladonato C., Ciccotti M.G., Tjoumakaris F.P., Freedman K.B. (2023). Combined Anterior Cruciate Ligament and Medial Collateral Ligament Reconstruction Shows High Rates of Return to Activity and Low Rates of Recurrent Valgus Instability: An Updated Systematic Review. Arthrosc. Sports Med. Rehabil..

[19] Lind M., Jacobsen K., Nielsen T. (2020). Medial collateral ligament (MCL) reconstruction results in improved medial stability: Results from the Danish knee ligament reconstruction registry (DKRR). Knee Surg. Sports Traumatol. Arthrosc..

[20] Funchal L.F.Z., Astur D.C., Ortiz R., Cohen M. (2019). The Presence of the Arthroscopic “Floating Meniscus” Sign as an Indicator for Surgical Intervention in Patients with Combined Anterior Cruciate Ligament and Grade II Medial Collateral Ligament Injury. Arthrosc. J. Arthrosc. Relat. Surg..

[21] Moher D., Liberati A., Tetzlaff J., Altman D.G., Group P. (2009). Preferred Reporting Items for Systematic Reviews and Meta-Analyses: The PRISMA Statement. Ann. Intern. Med..

[22] Hefti F., Miiller W., Jakob R.P., Stiiubli H.-U. (1993). Evaluation Evaluation of knee ligament injuries with the IKDC form. Knee Surg. Sports Traumatol. Arthrosc..

[23] Tegner Y., Lysholm J. (1985). Rating Systems in the Evaluation of Knee Ligament Injuries. Clin. Orthop. Relat. Res..

[24] Briggs K.K., Lysholm J., Tegner Y., Rodkey W.G., Kocher M.S., Steadman J.R. (2009). The Reliability, Validity, and Responsiveness of the Lysholm Score and Tegner Activity Scale for Anterior Cruciate Ligament Injuries of the Knee: 25 Years Later. Am. J. Sports Med..

[25] Higgins J.P.T., Thompson S.G. (2002). Quantifying heterogeneity in a meta-analysis. Stat. Med..

[26] IntHout J., Ioannidis J.P.A., Borm G.F. (2014). The Hartung-Knapp-Sidik-Jonkman method for random effects meta-analysis is straightforward and considerably outperforms the standard DerSimonian-Laird method. BMC Med. Res. Methodol..

[27] IntHout J., Ioannidis J.P.A., Rovers M.M., Goeman J.J. (2016). Plea for routinely presenting prediction intervals in meta-analysis. BMJ Open.

[28] Walker E., Hernandez A.V., Kattan M.W. (2008). Meta-analysis: Its strengths and limitations. Cleve Clin. J. Med..

[29] Halinen J., Lindahl J., Hirvensalo E., Santavirta S. (2006). Operative and Nonoperative Treatments of Medial Collateral Ligament Rupture with Early Anterior Cruciate Ligament Reconstruction. Am. J. Sports Med..

[30] Zaffagnini S., Bonanzinga T., Muccioli G.M.M., Giordano G., Bruni D., Bignozzi S., Lopomo N., Marcacci M. (2011). Does chronic medial collateral ligament laxity influence the outcome of anterior cruciate ligament reconstruction?. J. Bone Joint Surg. Br..

[31] Westermann R.W., Spindler K.P., Huston L.J., Wolf B.R., Amendola A., Andrish J.T., Brophy R.H., Flanigan D.C., Jones M.H., Kaeding C.C. (2019). Outcomes of Grade III Medial Collateral Ligament Injuries Treated Concurrently with Anterior Cruciate Ligament Reconstruction: A Multicenter Study. Arthrosc. J. Arthrosc. Relat. Surg..

[32] Tischer T., Geier A., Lenz R., Woernle C., Bader R. (2017). Impact of the patella height on the strain pattern of the medial patellofemoral ligament after reconstruction: A computer model-based study. Knee Surg. Sports Traumatol. Arthrosc..

[33] Yang X.-G., Wang F., He X., Feng J.-T., Hu Y.-C., Zhang H., Yang L., Hua K. (2020). Network meta-analysis of knee outcomes following anterior cruciate ligament reconstruction with various types of tendon grafts. Int. Orthop..

[34] Tashiro T., Kurosawa H., Kawakami A., Hikita A., Fukui N. (2003). Influence of Medial Hamstring Tendon Harvest on Knee Flexor Strength after Anterior Cruciate Ligament Reconstruction: A Detailed Evaluation with Comparison of Single- and Double-Tendon Harvest. Am. J. Sports Med..

[35] Fanelli G.C., Edson C.J. (2002). Arthroscopically assisted combined anterior and posterior cruciate ligament reconstruction in the multiple ligament injured knee: 2- to 10-year follow-up. Arthrosc. J. Arthrosc. Relat. Surg..

[36] Hammoud S., Reinhardt K.R., Marx R.G. (2010). Outcomes of Posterior Cruciate Ligament Treatment: A Review of the Evidence. Sports Med. Arthrosc. Rev..

[37] Guenther D., Pfeiffer T., Petersen W., Imhoff A., Herbort M., Achtnich A., Stein T., Kittl C., Schoepp C., Akoto R. (2021). Treatment of Combined Injuries to the ACL and the MCL Complex: A Consensus Statement of the Ligament Injury Committee of the German Knee Society (DKG). Orthop. J. Sports Med..

[38] Thorstensson C.A., Lohmander L.S., Frobell R.B., Roos E.M., Gooberman-Hill R. (2009). Choosing surgery: Patients’ preferences within a trial of treatments for anterior cruciate ligament injury. A qualitative study. BMC Musculoskelet. Disord..

[39] Diermeier T., Rothrauff B.B., Engebretsen L., Lynch A.D., Ayeni O.R., Paterno M.V., Xerogeanes J.W., Fu F.H., Karlsson J., The Panther Symposium ACL Treatment Consensus Group (2020). Treatment after anterior cruciate ligament injury: Panther Symposium ACL Treatment Consensus Group. Knee Surg. Sports Traumatol. Arthrosc..

[40] Razi M., Soufali A.P., Ziabari E.Z., Dadgostar H., Askari A., Arasteh P. (2021). Treatment of Concomitant ACL and MCL Injuries: Spontaneous Healing of Complete ACL and MCL Tears. J. Knee Surg..

[41] Spindler K.P., Wright R.W. (2008). Clinical practice Anterior Cruciate Ligament Tear. N. Engl. J. Med..

[42] Kim S.-J., Lee D.-H., Kim T.-E., Choi N.-H. (2008). Concomitant reconstruction of the medial collateral and posterior oblique ligaments for medial instability of the knee. J. Bone Joint Surg. Br..

